# The workplace masking experiences of autistic, non-autistic neurodivergent and neurotypical adults in the UK

**DOI:** 10.1371/journal.pone.0290001

**Published:** 2023-09-06

**Authors:** Amber Pryke-Hobbes, Jade Davies, Brett Heasman, Adam Livesey, Amy Walker, Elizabeth Pellicano, Anna Remington

**Affiliations:** 1 UCL Centre for Research in Autism and Education (CRAE), University College London, London, United Kingdom; 2 School of Education, Language and Psychology, York St John University, York, United Kingdom; 3 Neurodiversity Works, London, United Kingdom; 4 University College London, London, United Kingdom; SUNY Downstate Health Sciences University, UNITED STATES

## Abstract

Masking entails hiding or concealing one’s traits during social interactions. Research suggests that masking is particularly common for autistic people, though many non-autistic people also conceal aspects of their identity. Existing research has identified the key motivations and consequences of masking. No research to date, however, has considered how this might be affected by the social context in which masking is employed. This study compared the masking experiences of 285 autistic, 88 non-autistic neurodivergent and 99 neurotypical adults within a context in which masking is expected to be highly prevalent, namely the workplace. We used reflexive thematic analysis to explore the motivations, consequences, and contextual differences of workplace masking compared to other social contexts. Workplace masking was considered by participants in all three groups to be an adaptive response to a range of socially grounded workplace challenges and was usually employed as a strategy to safeguard against the threat of negative social and employment outcomes. Our non-autistic neurodivergent and autistic participants, however, reported experiencing unique pressures to mask, given the limited understanding of neurodiversity in workplaces and society more broadly. These findings have important implications for the wider masking literature and for workplace practice.

## Introduction

The Office for National Statistics [1] estimates that only 52.1% of disabled 16–64-year-olds in the United Kingdom (UK) are in paid employment, compared to 81.3% of their non-disabled counterparts. For autistic adults, this statistic is even lower, with a mere 21.7% reportedly in paid employment, the lowest of all disability categories [1]. This disparity in employment rates reflects the ongoing unemployment crisis in the UK for autistic adults. Employment has demonstrable benefits on autistic adults’ wellbeing, including an improved sense of purpose, quality of life, and physical health [2–4]. Given the prevalence of mental health conditions in the autistic population [5], greater efforts must be made to address this employment gap.

Notwithstanding that both employers and autistic employees recognise the desirable qualities that many autistic individuals offer in the workplace, such as reliability, creativity, and integrity [6,7], autistic adults continue to face a plethora of barriers in the search for paid employment [8–11]. One such barrier identified in recent research [8] is the stigma associated with autism–a barrier that more than 80% of autistic participants reported as challenging to overcome. Stigma, as defined by Goffman [12], refers to the social discrediting of individual or group attributes, resulting in individuals feeling unaccepted and rejected. Extant research investigating such attitudes in the UK indicates that autism-related stigma remains highly prevalent. For example, in a study by Wood and Freeth [13] in which non-autistic students (n = 42) were asked to rate how positive or negative stereotypical autistic traits were on a 7-point Likert scale, 80% of presented traits were reported to be perceived negatively.

For autistic adults who secure employment, research suggests that the stigma associated with being autistic persists as an employee [14–16]. As a result, once in employment, autistic employees are faced with the complex decision of whether to disclose their diagnosis to employers and colleagues. Indeed, many autistic employees are deterred from disclosing that they are autistic out of fear of being stigmatised by employers and colleagues [14,16–20]. Research by the National Autistic Society [15] indicates that this fear is not unfounded: more than half of autistic employees report being the recipient of workplace discrimination or bullying as a result of being autistic [15].

While disclosing one’s autistic status has been associated with negative stigma-related outcomes [14–16,21], research from outside of the employment field indicates that this label may not solely account for the stigmatisation of autistic people [22–24]. Specifically, studies have identified autistic people’s social communication behaviours during social interactions as a key source of stigma [9,22]. Initial research by Sasson and Morrison [24] found that first impressions of autistic adults, when rated by observers who were unaware of their diagnostic status, were less favourable than those of non-autistic adults, with observers perceiving autistic adults to be less approachable and more awkward than non-autistic adults. While non-autistic people’s first impressions were less negative in conditions where autistic adults’ autism diagnosis was disclosed [24], negative social evaluations of autistic adults appear to have adverse implications for future intentions, with non-autistic observers reporting that they would be less likely to initiate a conversation or pursue a friendship with an autistic adult [25]. Autistic employees’ reports of their experience in the workplace mirror this sentiment, detailing that differences in communication styles can be a barrier to making friends at work [26,27].

To avoid the negative social evaluations that stem from autism-related stigma, many autistic adults report employing arduous masking strategies [28–30]. Masking, also referred to in the autism literature as camouflaging, can be defined as the conscious or unconscious suppression of natural responses, accompanied by the adoption of alternative responses, across domains such as social interaction, sensory experience, cognition, movement, and behaviour [31]. In line with the preferences of the autistic community [32], we use the term masking, rather than camouflaging, throughout this paper. Yet, masking is not a strategy employed exclusively by autistic people [33]. People’s attempts to present themselves in ways that are acceptable to others, even to the detriment of their own well-being, have been acknowledged in sociology since at least the early post-war years [34]. Sociological research has also often connected these strategies to the deep structural injustices that blight the lives of a series of demographic groups. For example, attention has been paid to the practice of ‘passing’ in the African American population, especially during the period before civil rights, whereby individuals intentionally sought to convince others that they were of a different racial identity in order “to adopt specific roles or identities from which [they] would otherwise be barred by prevailing social standards” [35].

As with the case of racial injustice, the existing literature suggests that autistic masking is strongly motivated by the desire to avoid or compensate for autism-related stigma. Accordingly, a study involving 223 autistic adults found that self-reported masking behaviours were positively predicted by the degree to which participants perceived their autistic identity as stigmatised [36]. Participants’ masking behaviours were also associated with using individualistic (dissociating from other autistic people) and collective strategies (positively ‘redefining’ autistic people compared to non-autistic people) to circumvent the stigma associated with being autistic [36]. The authors explain this relationship between stigma and masking through the lens of Social Identity Theory [37]. On this account, to avoid the stigma associated with the autistic identity, individuals endeavour to obtain membership in a higher-status out-group by adopting the behaviours and traits of that group. In the case of autistic masking, to be accepted within neurotypical social groups, autistic people adopt behaviours associated with a neurotypical identity and conceal behaviours associated with their autistic identity. However, autistic people may also engage in collective strategies, such as joining the autistic rights movements, in order to positively re-conceptualise the perception of autistic people (in-group) compared to the neurotypical population (out-group) [36]. Although such identity shifts can enable autistic people to ‘fit in’ [28] and increase social contact [29], masking often has deleterious effects on one’s wellbeing [30,38–40]. Accordingly, autistic adults who regularly mask are at an increased risk of experiencing symptoms of depression, anxiety, and lifetime suicidality [28,41].

To understand better the adverse health consequences sometimes associated with masking, we need to consider the contexts in which these strategies are employed. In the only study to our knowledge to have explored the correlational effects of masking across social contexts, Cage and Troxell-Whitman [28] found that masking–both consistently and frequently across different social contexts as well as in some social contexts but not in others–was associated with a greater likelihood of stress in autistic participants than masking infrequently. Given the cross-sectional nature of this study [28], however, eliciting people’s subjective experiences of masking in particular contexts may reveal greater insights into the nuances in the relationship between masking and mental health. The current study therefore aims to examine the subjective experiences of masking within a context in which masking may be particularly salient–namely the workplace. Since existing research indicates that masking is particularly prevalent in formal social contexts, such as in the company of colleagues and employers, and significantly motivated by employment-based goals [28], we expect that an investigation of masking within this context is likely to reveal core experiences, motivations, and consequences associated with this behavioural strategy.

In the current study, we compared the first-hand workplace masking experiences of autistic, non-autistic neurodivergent and neurotypical adults, to (1) identify which experiences, if any, may be unique to a particular group and (2) establish how masking in the context of the workplace may differ from masking in other contexts. Neurodivergence refers to individuals with a different neurology to that of the general population [42]. Examples of neurodivergence include autism, dyslexia, dyspraxia, and attention deficit hyperactivity disorder [43,44]. For the purpose of this study, the term (non-autistic neurodivergent) relates to individuals who reported a formal or self-diagnosis of a neurodivergent condition (excluding being autistic) or a mental health condition. The grouping of participants in this way is not intended to suggest that being autistic is, or should be, distinguished from other neurodivergent conditions; nor is it intended to suggest that neurodivergent people can be conceptualised as one homogenous group. Nonetheless, by grouping the sample in this way, we can explore similarities and differences between different groups’ experiences of workplace masking. Moreover, despite ongoing discourse surrounding whether mental health conditions should be included under the umbrella of neurodiversity, we believe that the common stigmatisation of this group renders it appropriate to consider the experience of these conditions alongside other forms of neurodivergence [42,44,45].

## Methods

This study forms part of a broader research initiative exploring employment experiences in the UK using a bespoke survey: the Diverse Minds Survey. The Diverse Minds Survey was open between February 2019 and October 2021 and invited participants to complete a series of optional questionnaires or ‘modules’ regarding different aspects of employment, including workplace masking. To take part, participants needed to be aged at least 18 years and have experience of employment, or job-seeking, in the UK. Participants were recruited through three channels: (*1*) a social media callout; (*2*) the Autistica research network–a network of autistic people in the UK interested in participating in autism research, and (*3*) organisations that registered their interest in understanding more about neurodiversity and employment. The call for recruitment invited adults (aged 18 years and above) to take part in a study exploring (1) neurodivergent experiences in the workplace; (2) how neurodivergent workplace experiences compare to those without any diagnosis, and (3) experiences of enabling employment practices for everyone (i.e., neurodivergent and neurotypical people).

### Participants

A total of 581 participants navigated to the survey on workplace masking. Participants were classified into one of three groups based on their self-reported (clinical or self) diagnoses: (1) autistic (*n* = 300); (2) non-autistic neurodivergent (i.e., those who reported a neurodevelopmental condition other than being autistic or a mental health condition) (*n* = 109) and (3) neurotypical (i.e., those who reported no neurodevelopmental or mental health condition) (*n* = 172). Participants were excluded from the analyses if they did not respond to any of the questions on workplace masking. In total, 9.0% of the autistic sample *(n =* 27), 26.6% of the non-autistic neurodivergent sample (*n* = 29), and 65.1% of the neurotypical sample (*n =* 112) were excluded. Of the excluded participants, 92 (54.8%) indicated the questions on workplace masking were not relevant to them. There were no significant differences between non-autistic neurodivergent participants who did complete the survey, and non-autistic neurodivergent participants who did not. Similarly, there were no significant differences between neurotypical participants who did complete the survey, and neurotypical participants who did not. Fisher’s Exact Tests indicated autistic people who chose not to complete the questions on workplace masking were more likely to be male (p = .016), unemployed and looking for work (p = .013) and dissatisfied with their current employment status (p = .011).

The final sample comprised 411 participants: 272 autistic, 78 non-autistic neurodivergent, and 61 neurotypical adults (see Table 1). In recognition of the persistent challenges in accessing adult diagnostic services [46,47] both clinically and self-identified autistic adults were included. The majority of the autistic participants (*n* = 224, 82.4%) reported having a clinical diagnosis, with the remainder (*n* = 48, 17.6%) self-identifying as autistic. There were few significant differences in the demographic information between clinically-diagnosed and self-diagnosed autistic participants. The exceptions to this pattern were that formally diagnosed participants were significantly more likely to report being from a white ethnic background (Fisher’s Exact Test, *p =* .006), and a greater degree of satisfaction with their employment status (Fisher’s Exact Test: *p =* .041*)*, than self-diagnosed autistic participants. Given that group differences were limited, clinically diagnosed and self-identified autistic participants are considered together.

**Table 1:** Demographic characteristics of autistic, non-autistic neurodivergent and neurotypical participants.

Variable	Autistic (A) participants (*n* = 272)	Non-autistic neurodivergent (ND) participants (*n* = 78)	Neurotypical (NT) participants (*n* = 61)	Group comparisons
**Gender**				A < NT*, ND**
Men (including trans men)	89 (32.7%)	34 (43.9%)	33 (54.1%)	
Women (including trans women)	163 (59.9%)	43 (55.1%)	27 (44.3%)	
Non-binary	20 (7.4%)	0 (0.0%)	0 (0.0%)	
Prefer not to say	0 (0.0)	1 (1.3%)	1 (1.6%)	
**Age**				A > NT *** A = ND, ND = NT
18–25	34 (12.5%)	7 (9.0%)	9 (14.8%)	
26–35	57 (21.0%)	23 (29.5%)	13 (21.3%)	
36–46	66 (24.3%)	17 (21.8%)	15 (24.6%)	
46–55	80 (29.4%)	22 (28.2%)	22 (36.1%)	
56–65	32 (11.8%)	6 (7.7%)	1 (1.6%)	
66–75	3 (1.1%)	2 (2.6%)	0 (0.0%)	
76+	0 (0.0%)	1 (1.3%)	0 (0.0%)	
Prefer not to say	0 (0.0%)	0 (0.0%)	1 (1.6%)	
**Ethnicity**				A > NT*, A = ND, ND = NT
White British	217 (79.8%)	70 (90.9%)	53 (86.9%)	
Other White backgrounds	28 (10.3%)	3 (3.4%)	0 (0.0%)	
Mixed/Multiple ethnic groups	11 (4.0%)	1 (1.3%)	2 (3.3%)	
Black/African/Caribbean/Black British	2 (0.7%)	1 (1.3%)	2 (3.3%)	
Asian/Asian British	0 (0.0%)	1 (1.3%)	3 (4.9%)	
Other ethnic groups	3 (1.1%)	1 (1.3%)	1 (1.6%)	
Prefer not to say	0 (0.0%)	1 (1.3%)	0 (0.0%)	
**Education**				A < NT*, ND < NT***, A = ND,
Bachelor’s Degree	75 (27.6%)	24 (30.8%)	19 (31.1%)	
Master’s degree	64 (23.5%)	22 (28.2%)	32 (52.5%)	
A/AS Level^1^	27 (9.9%)	6 (7.7%)	1 (1.6%)	
Doctorate	19 (7.0%)	3 (3.8%)	2 (3.3%)	
GCSEs^2^	19 (7.0%)	4 (5.1%)	0 (0.0%)	
BTEC^3^	15 (5.5%)	5 (6.4%)	2 (3.3%)	
Higher National Diploma^3^	13 (4.8%)	0 (0.0%)	1 (1.6%)	
Post Graduate Certificate	11 (4.0%)	6 (7.7%)	0 (0.0%)	
Post Graduate Diploma	8 (2.9%)	2 (2.6%)	2 (3.3%)	
No formal qualifications	7 (2.6%)	2 (2.6%)	0 (0.0%)	
Foundation degree	6 (2.2%)	2 (2.6%)	1 (1.6%)	
GNVQ^3^	4 (1.5%)	1 (1.3%)	0 (0.0%)	
Professional qualification	4 (1.5%)	1 (1.3%)	1 (1.6%)	
**Employment status**				A < ND*, NT*, ND < NT***
Employed full time	102 (37.6%)	53 (67.9%)	49 (80.3%)	
Employed part time	60 (21.8%)	10 (12.8%)	11 (18.0%)	
Unemployed (looking for work)	22 (9.5%)	3 (3.8%)	0 (0.0%)	
Self-employed	26 (9.6%)	3 (3.8%)	1 (1.6%)	
Unemployed (not looking for work)	21 (7.7%)	1 (1.3%)	0 (0.0%)	
Student	13 (5.0%)	1 (1.3%)	0 (0.0%)	
Volunteer	12 (4.4%)	0 (0.0%)	0 (0.0%)	
Retired	7 (2.6%)	5 (6.4%)	0 (0.0%)	
Full time Parent/Carer	6 (2.2%)	0 (0.0%)	0 (0.0%)	
Unemployed (with independent means)	1 (0.4%)	0 (0.0%)	0 (0.0%)	
Apprentice or intern	1 (0.4%)	1 (1.3%)	0 (0.0%)	
Temporary employment	1 (0.4%)	0 (0.0%)	0 (0.0%)	
Employed (unspecified)	0 (0.0%)	1 (1.3%)	0 (0.0%)	
Prefer not to say	0 (0.0%)	0 (0.%)	0 (0.0%)	
**Income**				A < ND*, NT* ND < NT*
< £10,000	63 (23.2%)	6 (7.7%)	0 (0.0%)	
£10,000 - £19,999	66 (24.3%)	10 (12.8%)	1 (1.6%)	
£20,000 - £29,999	61 (22.4%)	13 (16.7%)	4 (6.6%)	
£30,000 - £39,999	26 (9.6%)	15 (19.2%)	20 (32.8%)	
£40,000 - £49,999	13 (4.8%)	14 (18.0%)	8 (13.1%)	
£50,000 - £59,999	6 (2.2%)	1 (1.3%)	3 (4.9%)	
£60,000 - £79,999	10 (3.7%)	5 (6.4%)	8 (13.1%)	
£80,000 - £99,999	3 (1.1%)	3 (3.8%)	6 (9.8%)	
£100,000 - £149,999	4 (1.5%)	1 (1.3%)	6 (9.8%)	
> £150,000	0 (0.0%)	0 (0.0%)	0 (0.0%)	
Prefer not to say	20 (7.4%)	3 (3.8%)	5 (8.2%)	
**Satisfaction with employment**				A*, ND* < NT
Satisfied	121 (44.5%)	42 (53.8%)	52 (85.2%)	
Uncertain	65 (23.9%)	14 (18.0%)	6 (9.8%)	
Dissatisfied	79 (29.0%)	17 (21.8%)	1 (1.6%)	
Prefer not to say	1 (0.4%)	2 (2.6%)	2 (3.3%)	
Not applicable	6 (2.2%)	3 (3.8%)	0 (0.0%)	
**Number of different employers**				A > NT*, ND > NT***, A = ND,
1–2	28 (10.3%)	17 (21.8%)	19 (31.1%)	
2–4	57 (21.0%)	17 (21.8%)	23 (37.7%)	
4–6	50 (18.4%)	15 (19.2%)	6 (9.8%)	
More than 6	128 (47.1%)	25 (32.1%)	10 (16.4%)	
None	7 (2.6%)	4 (5.1%)	2 (3.3%)	
Prefer not to say	2 (0.7%)	0 (0.0%)	1 (1.6%)	

* *p* < .001

** *p* = .002

*** *p = <* .*05*.

^1^ AS-A-Levels are qualifications in the UK that are typically taken between 16 and 18 years old.

^2^ GCSEs are qualifications in the UK that are typically taken between 14 and 16 years old.

^3^ BTECs, GNVQs and Higher National Diplomas are vocational qualifications in the UK. The last GNVQs were awarded in 2007. BTECs are approximately equivalent to GCSE and AS/A-Level qualifications. Higher National Diplomas are approximately equivalent to years one and two of a Bachelor’s Degree.

Just over half of autistic (*n* = 163 of 272, 59.9%) and non-autistic neurodivergent (*n* = 43 of 78, 55.1%) participants identified as women, while just under half of neurotypical participants (*n* = 27 of 61, 44.3%) identified as women. Fisher’s Exact Tests showed there were significantly more women in the autistic group than the neurotypical (*p* < .001) and non-autistic neurodivergent (*p* = .002) groups. Across the whole sample, there was a notable lack of ethnic/racial diversity. Specifically, 79.8% of autistic participants (*n* = 217 of 272), 90.9% of non-autistic neurodivergent participants (*n* = 70 of 78), and 86.9% of neurotypical participants (*n* = 53 of 61) reported being of a white British ethnic background. The majority of autistic participants (*n* = 234, 86.0%) reported a co-occurring condition, with anxiety (*n* = 100 of 234, 42.7%), unique sensory processing (*n* = 52 of 234, 22.2%) and ADHD (*n* = 35 of 234, 15.0%) being the most common. The most common diagnoses within the non-autistic neurodivergent sample included anxiety (*n* = 25 of 78, 32.1%), depression (*n* = 19 of 78, 24.4%) and dyslexia (*n* = 13 of 78, 16.7%).

Just over one third of autistic participants (*n* = 102 of 272, 37.6%) were in full-time employment compared to most non-autistic neurodivergent (*n* = 53 of 78, 67.9%) and neurotypical participants (*n* = 49 of 61, 80.3%). Significantly more neurotypical participants were in full-time employment than both autistic (*p* < .001) and non-autistic neurodivergent (*p* < .001) participants. More non-autistic neurodivergent participants were in full-time employment than autistic participants (*p* < .001). Neurotypical participants had a higher earning power than autistic (*p* < .001) and non-autistic neurodivergent (*p* < .001) participants, while non-autistic neurodivergent participants had a higher earning power than autistic participants (*p* < .001).

When asked if they were satisfied with their employment status, less than half of the autistic participants reported they were (*n* = 121 of 272, 44.5%), compared to more than half of the non-autistic neurodivergent participants (*n* = 42 of 78, 53.8%) and the majority of the neurotypical participants (*n* = 52 of 61, 85.2%). Significantly more participants in the neurotypical sample reported being satisfied with their employment status than in the autistic (*p* < .001) and non-autistic neurodivergent (*p* < .001) samples. See Table 1 for further group comparisons.

### Materials

The current study used a bespoke questionnaire, presented as part of the Diverse Minds Survey, a UK-wide online survey exploring the employment experiences of autistic adults. The Diverse Minds survey was created as part of Discover Autism in Research and Employment, a collaborative project between the UCL Centre for Research in Autism and Education and the UK autism research charity, Autistica.

All participants completed a section at the beginning of the survey on their demographic and employment data. This section contained questions regarding the participants’ gender identity, age category, ethnicity, and highest level of education as well as more employment specific questions (e.g., employment status, income, satisfaction with employment status, number of different employers). Participants were then presented with a number of survey modules on different topics in employment (e.g., see [18,48,49]). Participants in the present study completed the survey module on workplace masking experiences. This section of the survey opened by defining workplace masking as “a term that describes the strategies people use to fit in at their workplace” and provided examples of masking strategies. The definition of masking provided was deliberately narrow to ensure participants focussed on experiences of masking in the workplace, as opposed to masking in other contexts. Example masking strategies included references to individuals’ management of their appearance, social interactions, and/or natural behaviours in a workplace setting. Participants were asked a number of (quantitative) questions related to workplace masking followed by a series of open questions probing for information regarding (1) perceived motivations for masking, or not masking, in the workplace; (*2*) perceived advantages and disadvantages of workplace masking; and (*3*) ways in which masking in the workplace may differ to masking in other contexts. The current paper focusses on the qualitative responses from the open questions only. See Supplementary Materials for full list of survey questions.

### Procedure

Participants were provided with a link to the survey, hosted on Qualtrics. Here, participants provided informed consent and responded to a series of demographic and employment-related questions. Subsequently, participants were given the option to answer any of the seven modules of the survey that were relevant to them, one of which was on masking. The masking module took participants approximately 20 minutes to complete. Participants received no monetary or other compensation for their contribution to the research. Ethical approval for the study was granted by the UCL Institute of Education Ethics Committee (REC1149).

### Data analysis

Open-text data were uploaded to NVivo [50] software and analysed within an essentialist framework (reporting participants subjective reality, meaning, and experience) using thematic analysis [51,52]. We employed an inductive approach was employed whereby codes and subsequent themes were generated according to the content of the data. Data analysis was led by the first author who immersed themselves in the data by reading and re-reading the responses. Patterns from autistic participants were identified and data extracts were assigned initial codes. Codes were subsequently reviewed and organised into themes and sub-themes, with support from A.R. Two authors (A.P.H & J.D) independently coded the responses from non-autistic neurodivergent and neurotypical participants against the same coding framework, in order to identify similarities and differences in responses. A.P.H and J.D. met to discuss the fit of the coding framework for these data, resolve any discrepancies, and agree on the final set of codes and, subsequently, themes and sub-themes. A diverse group of non-autistic neurodivergent, autistic and non-autistic colleagues agreed on the final set of themes and sub-themes present across the dataset.

### Findings

**Fig 1:**
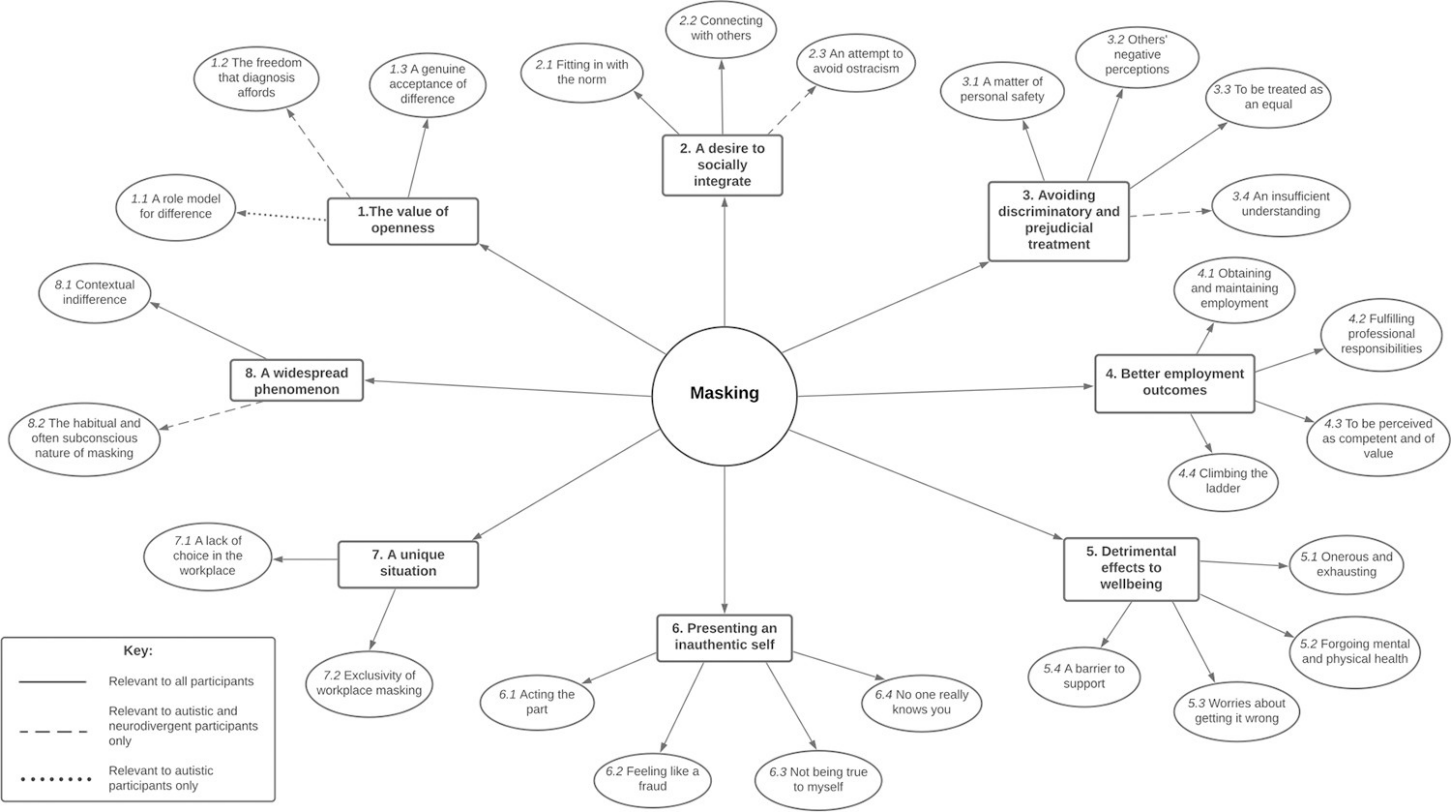
Thematic map of participants’ workplace masking experiences.

We identified eight themes from the qualitative data (see Fig 1). There was much overlap between the themes identified in the autistic, non-autistic neurodivergent and neurotypical datasets; the qualitative results are therefore presented together, below, and group differences are highlighted in the text with illustrative quotes. All quotes are accompanied by a participant ID indicating whether they are autistic (A), non-autistic neurodivergent (ND) or neurotypical (NT).

### Theme 1: The value of openness

#### 1.1 “A role model for difference”

Several autistic participants spoke of the responsibility they felt to be open and honest about their autistic identity in the workplace. Participants expressed a desire to unmask (i.e., stop concealing their true identity and traits) in order to set a positive example for other autistic colleagues and “make things better for the next generation” (A-397). ‘Unmasking’ was also referenced as a way for participants to exhibit their autistic strengths to non-autistic colleagues and improve the acceptance of autistic differences in the workplace: “if people know about your autism, [it] can help reduce stigma surrounding the condition” (A-309).

#### 1.2 The Freedom that diagnosis Affords

Both autistic and non-autistic neurodivergent participants recognised the importance of having a formal diagnosis when choosing to unmask in the workplace. Following a diagnosis, participants reported that they felt a reduced obligation to mask: “I honestly don’t really bother to mask–getting a diagnosis gave me permission to wear comfy shoes, stop making eye contact if it’s uncomfortable etc.” (ND-012). Although diagnosis gave participants the confidence to unmask, they also acknowledged the difficulty of doing so after masking for so long: “Since my diagnosis, I am trying to not mask as frequently. This isn’t easy. Sometimes, I cannot recognise that I’m masking in the first place” (A-432).

#### 1.3 A genuine acceptance of difference

The varying degree to which participants experienced acceptance and understanding of their differences was also referenced as a reason to selectively unmask. For example, one non-autistic neurodivergent participant commented that they did not mask as they were “not ashamed of being different” (ND-152). Most participants, however, attributed this acceptance externally, citing the positive role that friends and family members played in the decision to unmask: “Socially I don’t have to do it as much because my friends and family know who I am, that I’m autistic, and don’t expect me to be different or more” (A-409). However, participants rarely recounted this level of understanding in the workplace. As such, one neurotypical participant noted that “with work… I am more careful [to not unmask]” (NT-074). Even positive experiences were recognised as not being ubiquitous. One participant, as a result, expressed their worries about the prospect of changing employers in the future: “At [my organisation], colleagues were supportive when I once had a meltdown… I’m just scared of moving to another [organisation] in the future in case they don’t understand” (A-274).

### Theme 2: A desire to socially integrate

#### 2.1 Fitting in with the norm

Participants across all three groups spoke of masking as one way to achieve social inclusion at work: “In the workplace, you mask to fit in” (NT-085). Though most participants felt that masking their differences was essential to be perceived as ‘normal’, the extent to which they wanted to fit in varied across the sample. While some merely sought to blend in and avoid being noticed while at work, others expressed a strong desire to be included in their team: “I wanted to be part of the team and so put a huge amount of effort into at least seeming to be like them” (A-235).

Participants cited two sources of motivation to fit in within the workplace–the self and others. While these two sources of motivation appeared to be distinct from one another, some participants endorsed both when reporting their reasons for masking at work. Self-oriented motivations for masking were expressed by participants as a desire to achieve social acceptance: “you want to feel accepted as you already sometimes feel weird enough” (A-255). Conversely, when discussing motivations that were other-oriented, participants were driven to appear normal to make it “easier” (A-250) for their colleagues: “It makes moment-to-moment interactions easier [and] it keeps people happy” (ND-106). However, participants expressed that this level of acceptance was superficial and only served to benefit them in the short term: “Masking has no advantages except very short-term ones of apparently being accepted into a group” (A-286).

#### 2.2 Connecting with others

The desire to form connections with colleagues was a key motivation behind workplace masking. Participants felt that masking made them more relatable to other colleagues, enabling them to form friendships: “I got curiosity out of people and quite an emotional connection with them. This was unique for me” (A-324). Indeed, the ability to be “liked, popular, [and] engaging” (NT-065) was highlighted as an advantage of workplace masking by many participants.

While some participants felt that masking was necessary to maintain the relationships formed with colleagues (“I don’t want to ruin social relationships [by unmasking]”; ND-107), others only employed masking strategies during initial interactions. In this respect, masking was used by autistic participants as a temporary tool to initiate workplace relationships and to avoid colleagues perceiving them “through the lens of autism” (A-435): “The advantage of masking is that people do not take an instant dislike to you, so it is possible over time… to get those who work with you to understand you before they pre-judge you based on their concept of acceptable behaviour” (A-463).

#### 2.3 An attempt to avoid ostracism

Without masking, autistic and non-autistic neurodivergent participants felt that they were at risk of social rejection within the workplace: “Obviously everyone has to filter themselves a bit at work, but I feel if I don’t do the right balance of masking, just being myself will end up getting me pushed away, alienated and kicked out” (A-294). Participants frequently drew on their previous experiences of marginalisation, both within and beyond the workplace, to inform their decision to mask around colleagues: “I always felt like an outsider and struggled to fit in” (ND-023). In an effort to avoid social rejection, autistic participants in particular emphasised the importance of hiding their true selves while working: “I worry that I will get shunned if I act myself and chat about my own interests, so I try to go along with what other staff are chatting about” (A-290).

### Theme 3: Avoiding discriminatory and prejudicial treatment

#### 3.1 A matter of personal safety

Participants in all groups were concerned that they may be subject to workplace “abuse [and] bullying” (ND-003) if they failed to mask: “In my previous workplace, I was less aware of my difficulties and therefore masked less–and was bullied almost to extinction” (A-266). As many attributed this bullying to their perceived differences, masking reportedly worked as an effective strategy to reduce this risk. The strict social expectations shared amongst colleagues, participants felt, fostered a culture of bullying within the workplace which targeted those who did not adhere: “The unwritten rules of social interaction and NT [neurotypical] etiquette in the workplace are rigid and expected. If you deviate, you’ll be… bullied” (A-397). In response, participants felt that masking enabled them to blend in sufficiently with their colleagues and avoid the bullying which resulted from being different: “The more noticeable you are the more open to bullying you are” (NT-134).

#### 3.2 Others’ negative perceptions

Masking was frequently employed by participants as a strategy to avoid the negative judgements formed by colleagues and employers: “I worry that I will be judged [if I don’t mask]” (NT-076). For many autistic and non-autistic neurodivergent participants, masking was employed to avoid the stereotypical beliefs that colleagues and employers held about their condition: “I have OCD and I don’t want my senior managers to make judgements based on that” (ND-057). A source of judgement that was commonly described amongst autistic participants was the discrepancy between their intentions and others’ interpretations of their behaviour, which often resulted in them being perceived as ‘rude’, ‘weird’ or ‘difficult’: “Once a customer misinterpreted my lack of eye contact as a dirty look… If I’d been masking maybe it wouldn’t have happened” (A-190).

#### 3.3 To be treated as an equal

Participants shared instances in the workplace during which they felt they had been treated as ‘less’ or ‘disabled’ because of their differences: “I worry about being pitied and being kept out of the loop. I know people are often more nervous after I disclose, less willing to take on board something I’ve said or laugh at a joke” (A-322). Participants felt that masking enabled them to circumvent this issue, allowing them to be treated without bias both socially and professionally in the workplace (“I don’t think I am viewed as the Dyslexic, I am viewed for what I can contribute”; ND-082) and be “seen just as me” (NT-118).

#### 3.4 An insufficient understanding

A lack of knowledge about neurodiversity amongst colleagues and managerial staff meant that autistic and non-autistic neurodivergent participants felt that masking was necessary to avoid “embarrassing conversations” (A-265) in the workplace. Indeed, masking was used by several participants as a strategy to avoid the burden of educating colleagues and employers about their neurodivergent experience:

I mask because I do not feel comfortable explaining my condition to people. The general perception of ADHD is that it is ‘just’ hyperactivity, and since this is not one of my main symptoms I find people can be dismissive or struggle to understand (ND-097).

As a result, many participants highlighted a need for colleagues and employers to learn more about neurodiversity, though an absence of open discussion in the workplace made that difficult: “I feel that my condition is known but never explicitly mentioned. This precludes any discussion of how it manifests and its impact on my daily life” (A-385). In addition, some autistic participants felt that while their organisation purported to support their autistic employees, this was often not translated into practice: “Despite working for an organisation that supports autistic people, I do not feel confident that they would understand” (A-330).

### Theme 4: Better employment outcomes

#### 4.1 Obtaining and maintaining employment

For many participants, their ability to secure and sustain employment was felt to be intrinsically linked to their ability to mask successfully in the presence of employers. The financial pressure that participants felt to maintain employment strongly influenced their decision to mask: “the risks of being different [at work] can effectively change your life as they are linked to your income” (ND-031). In many cases, this pressure was perceived as more important than the personal expenses incurred due to masking: “I value being able to sustain myself over my ability to be comfortable at work” (A-279)–and participants frequently shared their worries about the financial ramifications of not masking in the workplace. As a result, many emphasised the “higher stakes” (ND-023) that were involved in workplace masking: “The need to look professional and holding down the job means that people are less likely to expose all their thoughts in the workplace. In other areas of social life, people may not be so worried about how they are perceived” (NT-109).

#### 4.2 Fulfilling professional responsibilities

When distinguishing between masking contexts, participants described upholding a standard of professionalism as an important part of masking in the workplace: “I find masking in the professional environment more difficult, but to me it feels more essential. As I work in a professional services environment, we need to provide a service” (ND-035). Indeed, participants believed that masking was essential to fulfil the responsibilities of their job role, such as “being polite, listening and turn taking” (A-189). For many, the ability to adequately meet professional demands heavily relied on effective communication with others in the workplace: “in the workplace… engagement and interaction is vital to the success of my role and the wider business” (NT-084). As a result, participants experienced “better working relationships” (NT-134) that were more cooperative and collaborative when masking, enabling participants to work more efficiently on shared tasks. However, many autistic participants expressed their frustration concerning the social proficiency required to do their job, sharing that “if I couldn’t fit in, I found my job harder and harder to do” (A-301).

#### 4.3 To be perceived as competent and of value

Participants reported engaging in workplace masking strategies in order to be perceived as a “respected and trusted member of the team” (NT-076). Masking allowed participants to manage their difficulties without compromising how others perceived them professionally. In particular, anxiety relating to the social responsibilities of participants’ job roles was an issue that many masked from colleagues and employers: “I mask that I have anxiety issues. I think it could be seen as a weakness and my colleagues would think I’m not as capable” (ND-048). While some participants used masking to maintain others’ perception of their competency, others felt that masking acted as a prerequisite to being considered capable in their job role. Participants felt that, in the eyes of employers, their non-typical behaviours would undermine their ability to meet the demands of their job: “if I were to be completely natural I would worry people’s perceptions of my behaviour may affect their trust in me to perform my duties and take on responsibilities” (A-288).

#### 4.4 “Climbing the ladder”

Developing a successful career was an important milestone indicated by participants, and one that many felt was only achievable by masking: “I mask in the workplace to protect my position. I think disclosing my diagnosis could reduce opportunities, slow down career progression, and effect relationships” (ND-048). Indeed, masking was felt to afford faster professional development: “[I mask] to get chartered and promoted quickly and take on more responsibility than others in my grad intake” (NT-076). Participants also expressed the belief that career progression was limited to mainstream pathways that heavily rely on conforming to typical norms and behaviours: “Things like projects and promotions often rest on emotional considerations like popularity or ‘seeming normal’, even in companies who claim that they do not” (A-457). In turn, participants often attributed their professional success in the workplace to their ability to mask successfully. However, some were resentful of this, commenting that they felt “trapped into masking” (A-351) by a desire to further their career.

### Theme 5: Detrimental effects to wellbeing

#### 5.1 Onerous and exhausting

Extreme exhaustion was almost ubiquitously described amongst participants as a harmful consequence of masking. For many, masking in the workplace involved maintaining their mask for long periods with little to no respite. As a result, many commented that while masking “may have some short-term benefit in infrequent, short or one-off encounters [but it is] too much effort to maintain over the longer term” (NT-145).

Comparing professional and social masking, participants described the relentless fatigue they were subject to in the workplace, commenting that “in other areas of life you can take a break, or stop and listen to music for a bit. In work you have to mask constantly” (A-209). Many participants felt that the quality of their work suffered due to the energy and effort that was necessary to mask: “Masking sacrifices my abilities; I hear less, I miss things, I burn more energy, and I cannot use my mind in ways that I know I can do very well” (A-234). This extreme depletion of resources also had negative implications for participants’ personal lives, with relationships with partners, friends, and family members worsening as a result of the exhaustion they experienced from masking: “[The] disadvantage [of masking] is I am exhausted by the end of the day, I don’t want to see anyone else and I don’t want to go outside on weekends” (ND-182).

#### 5.2 Forgoing mental and physical health

Participants experienced worse health, both physically and mentally, as a result of masking in the workplace. Masking an array of sensory difficulties, such as harsh artificial lighting and repressed stims, often left participants feeling physically unwell:

At the end of a shift, I was so confused, frightened, overwhelmed, and anxious etc my head was so full and loud and busy with absolutely everything that had happened–all the smells, sounds, people, clothes, conversations, lights, telephones, paperwork, movement, voices, hustle and bustle of people etc. my brain constantly replaying everything I had smelt, felt, seen, heard etc. my head felt dizzy, with a high pitched ringing sound (ND-003).

When sharing the emotional costs of masking in the workplace, participants frequently described symptoms of chronic stress, depression and anxiety. Masking their true selves consistently at work created feelings of inadequacy about who they really were: “you feel your real self is worthless because if it wasn’t you wouldn’t have to pretend to be someone else” (A-423). However, participants felt that as their mental health worsened, the pressure to mask only increased, further exacerbating their deteriorating health: “You become overly concerned with how others perceive you [which] introduces more stress, which in itself becomes something you try to mask. A bit of a vicious circle” (NT-065). This growing pressure to mask while also managing their worsening mental health often resulted in participants experiencing meltdowns, shutdowns, and burnout: “[Masking] takes a lot of energy and I quickly burn out” (A-126). Consequently, many participants, reported taking extended sick leave or leaving their job permanently.

#### 5.3 Worries about getting it wrong

Unsuccessful attempts to mask were a source of concern, including concerns about whether their efforts to imitate others’ behaviour were convincing enough to go unnoticed by colleagues: “[There’s a] constant mental battle of coping with ‘what ifs’, unhelpful thinking patterns, and catastrophising that can co-occur when feelings of ‘masking gone wrong’ appear” (A-288). These worries extended to participants’ fear about the mask slipping unintentionally. Several participants described the embarrassment experienced during situations in which their mask had slipped and worried about the “reaction when the ‘mask’ is discovered” (NT-118) again in the future. These concerns, participants indicated, were more prominent following workplace masking, rather than social masking, as the consequences of getting it wrong were perceived as more costly. For many, the rumination that followed experiences of masking in the workplace meant that they “can never truly relax… I am always on edge about how people may perceive my actions. It can cause me to replay situations in my head which I wish had gone better” (ND-117). Even outside of the workplace, these worries continued to pervade participants’ thoughts, with one participant reporting that their mind continued “running over the day, processing it, [and] looking for hidden meanings” (A-282), which prevented them from being able to relax at home.

#### 5.4 A barrier to support and understanding

Participants felt that masking in the workplace precluded them from seeking and receiving support from employers that might otherwise be available if they unmasked. This was reflected in participants’ inability to access workplace adjustments: “You can’t have [the] adjustments you need so you have to endure sensory overload and all manner of other difficulties” (A-390). Masking such difficulties projected an inaccurate impression that participants were coping in the workplace, leaving other members of staff ignorant to their daily challenges: “People did not understand how I was feeling [because I was masking]” (NT-047). As a result, participants’ difficulties were often undetected by others for long periods of time: “By masking… I think it took a lot longer before someone realized that I did have a problem and was able to point me in the right direction to get help” (ND-138). These false impressions also had negative implications for participants who decided to disclose in the workplace, with autistic participants reporting that colleagues perceived them to be ‘less autistic’ as a result: “the more you mask and the better you are at doing it, the more people can’t equate autism and you together and completely dismiss any difficulties you may have” (A-332).

### Theme 6: Presenting an inauthentic self

#### 6.1 Acting the part

When sharing their experience of masking across contexts, participants often described it to be analogous to the role of a performing actor. The role that participants performed in the workplace, however, was one many reported as being distinctively disparate from their true selves: “Masking allows you to adopt a persona and live through that persona, whether it is close to your true character or not” (NT-168). Nonetheless, this ability to adapt their persona across different situations was a source of pride for many participants, commenting that it enabled them to be more confident in situations that they otherwise wouldn’t be: “even if you’re not comfortable with something you can take on a persona that is. [Masking] allows you to go outside your comfort zone and reach new heights” (ND-089).

When differentiating between masking contexts, some participants noted that adapting their mask to accommodate different situations was easier in the workplace than in social settings. The well-defined professional expectations outlined in formal regulations and procedures meant that some autistic and non-autistic neurodivergent participants found masking in the workplace easier to implement than during social interactions: “I find [workplace masking] easier as there’s a certain predictability in a business environment and with the right knowledge you can basically learn the script for your role” (A-233). In contrast, social masking was perceived to be more unpredictable in nature, leaving some participants feeling “more at sea out of work” (A-344): “If friends gave me a written contract and ‘friend job description’ maybe I would be better at social interaction” (A-390).

#### 6.2 “Feeling like a fraud”

Presenting a false persona at work often left participants feeling guilty about deceiving their colleagues and employers: “it feels dishonest, and I don’t like trying to be someone I am clearly not” (A-195). As a result, many described the acceptance they experienced within their working team as undeserved and granted under false pretence. This left participants with worries about others recognising their dishonesty and being perceived as “two-faced” (A-432) and a “charlatan” (A-401). Others, in contrast, reported that the mixed messages that masking communicated to their colleagues, had tangible negative effects on their working relationships: “[You’re] less honest with your colleagues, [so] they find you less approachable [and it’s therefore] harder to build up rapport with colleagues, making teamwork harder” (NT-158). One participant felt that these difficulties were a result of the inauthentic messages which masking communicated to others: “I think sometimes masking creates a confused message to people because it is important to be genuine” (ND-005).

#### 6.3 “Not being true to myself”

The benefits of masking were often described by participants as a trade-off for being oneself. For many, masking inherently discouraged them from being their authentic selves in the workplace, and instead encouraged them to present a persona congruent with employers’ expectations: “I felt I was looking out of a body that was not mine. In work I had to pretend to be someone who was very far removed from who I believed I was” (ND-003). Consequently, participants described a weak sense of belonging in their working team: “I find any aspect of pretending to be someone that I’m not to be damaging, [it] just makes me feel worse and [I] still have feelings of being an outsider” (NT-146). Routinely presenting this false persona in the workplace also had negative implications for participants’ sense of identity. In particular, many reported that they had lost the confidence to be themselves as a result of masking and frequently questioned who they truly were without the mask. “I sometimes feel like a Russian doll–there are so many layers but when they are all stripped away is there anything of the real me left inside?” (A-307).

#### 6.4 “No one really knows you”

Masking in the workplace undermined participants’ desire to be authentically understood by other colleagues: “some people in my life only see me masking so I sometimes feel people don’t know me as well as they think they do” (ND-186). Unable to develop genuine connections with others, participants were often left feeling socially disconnected in the workplace: “it is a slightly isolating experience. I see some people forming life long friendships, while I have worked with some people for 17 years, and never really became good friends” (NT-094). Of those participants who did develop friendships in the workplace, many expressed a dissatisfaction that these were grounded in their masking persona rather than their true self: “people don’t get to know the real you, so any friendship is based upon them liking your fake persona, not you” (A-432). This disparity between participants’ true self and others’ perceptions of them led a number of participants to doubt whether colleagues would accept them if they chose to unmask in the future.

### Theme 7: A unique situation

#### 7.1 A lack of choice in the workplace

According to participants, a key difference between workplace and social masking was the extent to which they felt that they had a choice in the decision to mask. In social contexts, participants felt an enhanced sense of personal control concerning the frequency and length of masking, commenting that “I can opt out of situations which I find challenging or walk away if I begin to feel over stimulated” (NT-074). In the workplace, however, participants felt professionally bound to mask throughout the working day: “It is not always possible to have space [in the workplace] when I need it. In my social life I don’t have the same restrictions and can decide when enough is enough” (A-323). Moreover, in social contexts, participants were able to choose company with whom they shared commonalities, commenting that this often was not the case with colleagues.

You also have no control over the colleagues you work with. In one’s social life you are able to choose the people you spend time with, and if you can’t be yourself around them you don’t have to spend time with them. Whereas you spend the majority of your time at work and you are not in control of who your fellow employees are and their personalities (ND-105).

#### 7.2 Exclusivity of workplace masking

For some participants, masking was a strategy only employed in the context of the workplace, reporting that “social life masking is not required” (NT-131). Socially, participants surrounded themselves with intimate friends and family, with whom they felt able to be themselves: “With family I can be myself and, as I have only very few true friends, I can also be myself with them even when that means being unsociable or uncommunicative–something that is not possible at work” (A-260). Others reported that they did not pursue social interactions outside of the workplace: “I do not mask in other areas of ‘social life’ as I do not consider that I have anything that I would describe as ‘social life’” (ND-181).

### Theme 8: A widespread phenomenon

#### 8.1 Contextual indifference

When asked to differentiate workplace masking from social masking, several participants expressed that there were no differences between these contexts as they “mask in all situations” (NT-158). Indeed, some participants described masking as invariant across both social and professional contexts: “It isn’t [different] really, it’s always playing a part, pretending to be neurotypical, remembering to keep eye contact, finding ways around my inability to hear with background noise, keeping a straight face when I’m screaming inside” (A-226). As such, while the demands of the social environment changed, the overarching masking strategies employed across contexts remained constant. “I’m not sure I am any different whether it’‘ in a professional environment or not, [the] same principles apply” (ND-089).

#### 8.2 The habitual and often subconscious nature of masking

Masking was described by many autistic and non-autistic neurodivergent participants as an automatic process that served as a daily coping strategy throughout their life. As a result, participants were often not cognisant of the routine masking strategies that they employed, commenting that it had become “second nature” (A-287) to them after so long: “Masking is something I have done for most of my life without even realising, since my diagnosis I have come to realise that masking is something I have done increasingly over the years at work to make life easier” (ND-186). Several autistic participants related this lack of meta-awareness of masking to their late diagnosis. This, in turn, left participants feeling that masking had become an integral part of who they were with no choice but to mask: “by the time you get to 47 and didn’t even know you were autistic until you were 45, [masking] is such a habit as to really not be a choice” (A-286).

## Discussion

To our knowledge, this is the first study to examine the workplace masking experiences of autistic, non-autistic neurodivergent and neurotypical adults. Our findings suggest that masking strategies are employed widely within the workplace by both neurodivergent and neurotypical employees. As such, our findings challenge popular definitions of masking that frame it as a strategy employed exclusively by autistic individuals [28,29,53; though see 30]. Rather, we present findings that indicate masking might be more accurately defined as a common experience among many non-autistic neurodivergent, and to some extent, neurotypical individuals. Indeed, the comparative nature of this study allowed us to identify a range of qualitative similarities and nuances in the workplace masking experiences of the three groups. For example, there was overlap in participants’ reasons for masking in the workplace, as well as their perceptions of the advantages and disadvantages of masking in this context. There were also, however, critical aspects of workplace masking that distinguished the experiences of our autistic and non-autistic neurodivergent participants from those of our neurotypical participants. For example, while autistic and non-autistic participants reported being motivated by many of the same goals as neurotypical participants, they also reported masking as a means by which to compensate for the lack of knowledge and awareness that others possessed about both being autistic but also about being neurodivergent more broadly. Moreover, when discussing masking itself, autistic and non-autistic neurodivergent participants reported unique challenges when attempting to suppress stimming behaviours and sensory sensitivities while at work. Next, we discuss our findings in relation to existing research and provide recommendations for how employers can support their employees who mask at work.

Our participants identified two key motivators for masking in the workplace. First, they highlighted the importance of social goals such as fitting in and making social connections with colleagues. While this finding could have been influenced by the given definition of masking (i.e., that it encompasses different strategies used to fit in), this finding is consistent with existing literature pertaining to the motivations for autistic and non-autistic masking (e.g., [28,29,33]). Importantly, autistic participants’ use of masking strategies to form and sustain relationships with their colleagues also challenges conventional assumptions that autistic people do not desire social relationships (see, for example, [54,55]). While participants in all groups expressed a desire to integrate successfully in the workplace, autistic and non-autistic neurodivergent participants reported unique concerns regarding the potential ostracism that they would face if they did not mask in the workplace. Indeed, both autistic and non-autistic neurodivergent participants expressed that an insufficient understanding of neurodiversity in the workplace further exacerbated the perceived pressure to mask in this context.

The second key motivator for workplace masking, common to all participant groups, was centred around a desire to gain and sustain fruitful and meaningful employment. In fact, our participants felt that masking was integral to successfully obtaining employment, fulfilling professional responsibilities, and developing professionally. Taken together, it can be considered that masking was employed as an adaptive response to a range of socially-grounded workplace challenges and was employed to safeguard against the threat of negative social and employment outcomes. This is in line with Goffman’s notion of “passing for normal” [56], in which people use ‘disidentifiers’ or choose not to disclose ‘discrediting information’ about themselves in order to pass as another, more accepted identity. In the context of this study, participants reported hiding their autistic or non-autistic neurodivergent identity, or indeed traits that were perceived as less accepted, to pass as neurotypical, and thus afford more positive social interactions and successful employment outcomes such as gaining and sustaining employment, or ‘climbing the ladder’.

Of course, individuals should not be expected to change who they are in order to succeed professionally–especially given the vast negative impacts masking can have on ones’ mental and physical wellbeing [28,30,38–41]. Instead, employers should endeavour to create an inclusive working culture in which employees feel confident to express their true selves, should they wish to. One way to develop such a culture may be through a programme of diversity and inclusion education which seeks to improve employees’ understanding of both neurodiversity and workplace masking. Indeed, evidence suggests that improvements in knowledge are associated with lower levels of stigma and improvements in first impressions [24,57–59]. As such, training on neurodiversity, with a specific focus on workplace masking, could result in a more inclusive workplace culture in which individuals feel less pressure to mask at work. While such training is likely to be beneficial for all employees, preliminary research within the employment literature indicates that the delivery of neurodiversity training may be particularly effective when targeted at those in supervisory and managerial roles [60,61]. Where possible, such training should also be co-produced with (autistic and non-autistic) neurodivergent individuals within the organisation, or with external neurodivergent collaborators, to ensure that they accurately represent the views and experiences of neurodivergent people and lead to meaningful change [62,63]. Another, perhaps more cost-effective, method to distribute information organisation-wide may be to develop short, easy-to-read guides outlining the different reasons for which masking might be used (e.g., maintaining professionalism, building confidence in one’s role, and/or avoiding stigma) and the possible consequences that masking may have for employees using these strategies. As the findings from both the current study and existing literature shed light on the potentially damaging effects of masking which include poorer mental health, increased stress and greater suicidal ideation [28,39,41], we hope that in educating organisations, both employers and colleagues will be better equipped adequately support those who mask at work. Future research should seek to evaluate the effectiveness of such materials.

As workplace masking was highly utilised in our sample, it is unsurprising that our participants highlighted a range of perceived advantages of masking in this context. Often, such advantages were grounded in the fact that masking enabled individuals to adhere to the social expectations and norms of the workplace. For many participants, these social expectations related to the specific context of the workplace. For example, some participants reflected that masking not only enabled them to appear professional in the company of colleagues, but also granted them with the confidence to better fulfil their roles. As a result of these workplace-specific factors, some participants did not report the need to mask in other social contexts. Yet, for other participants, the desire to conform with social expectations extended to contexts beyond that of the workplace, meaning they felt unable to unmask professionally *or* socially. Nonetheless, masking at work enabled participants to follow mainstream career paths and circumvent the stigma and discrimination to which they may otherwise be subject to.

Despite workplace masking being functionally adaptive, our findings also indicate that workplace masking can be associated with a range of negative consequences. For example, symptoms of extreme exhaustion, anxiety, and disconnection with one’s identity were commonly reported as a consequence of workplace masking. While these findings are consistent with previous accounts of masking [29,33], this study is the first to consider if the consequences of masking differ according to context. Indeed, the current results highlighted some phenomenological differences between participants’ experience of workplace masking and masking in other contexts. For example, some participants reported that the professional standards expected of employees within the workplace allowed for masking strategies to be implemented with greater ease and confidence than was possible in social contexts. In contradiction such reflections, however, the current study also highlighted cases in which participants felt that the increased intensity and regularity of workplace masking, compared to masking in other contexts, resulted in more extreme exhaustion and faster burnout. In addition, a reduced feeling of control over the decision to mask in the workplace, compared to the decision to mask socially, was a common contextual distinction reported by participants. These critical contextual differences suggest that workplace masking may have more severe ramifications for individuals’ mental health and wellbeing than masking in other contexts. While improvements in workplace culture may reduce the need to mask, it is unlikely that workplace masking can be entirely eliminated. As such, employers should seek to prioritise employees’ mental health by providing adequate levels of mental health support within the workplace. This support could be offered in the form of employment benefits (e.g., access to occupational therapy, or private counselling), reasonable adjustments (e.g., by allowing employees to work from home where necessary) or by more internal means (e.g., distributing relevant mental health resources, via a company intranet page). Future work should seek to evaluate the support that is currently available for employees who mask at work.

Finally, our findings suggest that participants’ ability to unmask in the workplace may, to some extent, be contingent on their diagnostic status. Indeed, participants who did not possess a formal diagnosis of a condition did not report experiencing the same freedom to unmask as participants with a formal diagnosis. Research with autistic adults supports this notion. For example, Lewis [64] reported that receiving a formal autism diagnosis during adulthood enabled participants to uncover previously hidden parts of themselves. Yet, requiring employees to possess a formal diagnosis to unmask in the workplace is problematic for several reasons. First, the process for obtaining some diagnoses (e.g., autism) can often be long, costly and fraught [44,64]. As such, it may not be possible for all people to access a formal diagnosis. Second, freedom in this respect is contingent on disclosing one’s diagnosis–something which many autistic and neurodivergent adults may not feel comfortable doing [18,19]. Third, our results indicate that masking is not exclusively employed by individuals who are, or think they may be, neurodivergent or autistic. As such, organisations should endeavour to create a culture that embraces difference and supports employees regardless of their diagnostic status.

### Limitations

There are several limitations to this study. First, it is important to note that our sample was self-selecting in nature. As such, it is possible that the participants in this study were those who were aware of their masking strategies and had an interest in sharing their experiences. Similarly, many, largely neurotypical participants, who indicated masking was not relevant to them, did not complete the survey. As such, we were unable to compare the experiences of individuals who *do* mask in the workplace with the experiences of those who *do not*. Of the neurotypical participants who were able to share their experiences of masking, we cannot be certain that their experiences reflect those of the wider neurotypical population, especially since our neurotypical respondents were likely to have prior awareness and knowledge about autism and/or neurodiversity owing to our recruitment strategy. In light of these sampling issues, future research comparing the masking experiences of different neurotypes should endeavour to recruit neurotypical participants with a range of knowledge about autism or neurodiversity.

Second, our sample is not likely to be demographically representative of the wider autistic, non-autistic neurodivergent or neurotypical populations. For example, given the nature of the data collection technique employed (an in-depth online survey), it is likely that some people with intellectual disability/ies were excluded, which means that we cannot speak to the workplace masking experiences of these individuals. In addition, there was an overrepresentation of individuals from a white British background across all groups. Given that research suggests that individuals from other minority ethnic groups experience unique pressures to mask aspects of their identity in the workplace [see, for example 64,65], it is possible that our findings understate the workplace masking experiences of individuals from minority ethnic groups. As such, future research should seek to examine the workplace masking experiences of individuals from minority ethnic backgrounds to establish whether the findings from the current study are relevant for these groups. Similarly, asymmetries in the gender distribution between our autistic, non-autistic neurodivergent, and neurotypical groups raises potential concern regarding the validity of our cross-group comparisons. // Indeed, our autistic sample contained significantly more women than both our non-autistic neurodivergent and neurotypical participant samples. This is despite current estimates suggesting a 3:1 male to female ratio regarding autism diagnoses [66]. While the gender distribution pattern of autistic participants in the current study is not unusual for survey-based research (see for example [67,68]), it is possible that the apparent differences in experiences of workplace masking across our groups were confounded by gender. Particularly, as previous research indicates that women tend to employ more masking behaviours than men [69–72], comparisons including our autistic group may have been affected by the high proportion of women included in this sample. Regrettably, however, due to a lack of non-autistic neurodivergent and neurotypical participants who identified as non-binary in the current study, our sample was not sufficiently representative to run such gender comparisons.

Third, the definition of masking provided in the current survey may have limited the range of experiences shared by participants. Particularly, by specifying the purpose of masking to ‘fit in’ rather than as a broad set of behaviours suppressing one’s natural responses, we may have failed to recruit participants for whom masking served a different function. Moreover, we did not ask our participants to define what workplace masking meant to them, which means we cannot be sure that the behaviours used to achieve the goals of workplace masking are comparable between different neurotypes. That said the commonalities in the experiences of workplace masking, especially in autistic and non-autistic neurodivergent participants, provide some confidence that their conceptualisation of workplace masking was similar. Nevertheless, future research should investigate the behavioural nuances in workplace masking between neurotypes to understand precisely how autistic and non-autistic neurodivergent people may be disproportiately affected.

Finally, we only examined the experiences of individuals with concealable stigmatised identities (including autism, other neurodevelopmental conditions and mental health conditions). As such, we cannot make any assumptions regarding the workplace masking experiences of individuals with visible differences (e.g., physical disabilities) based on our findings. While literature does suggest that individuals with visible differences engage in masking behaviours [73–76], it is likely that these experiences differ somewhat to the experiences of individuals who are able to, to some extent, conceal their true identity. Future research may seek to compare the workplace masking experiences of individuals *with* concealable identities to those *without* concealable identities to establish whether their experiences differ.

## Conclusion

This study identifies the contextual intricacies of masking in the workplace. While workplace masking was a common experience for autistic, non-autistic neurodivergent *and* neurotypical participants, masking in this context was not without its consequences. Indeed, many of our participants highlighted that workplace masking was a more frequent experience than masking in other contexts and was perceived to have more severe ramifications for their mental health. Our non-autistic neurodivergent and autistic participants reported experiencing unique pressure to mask, given the limited awareness of neurodiversity within workplaces and society more broadly. To improve experiences, we suggest that employers should endeavour to (1) foster an accepting working environment in which employees do not feel a pressure to conceal aspects of their identity using masking strategies, (2) provide frequent, comprehensive training on neurodiversity and masking, and (3) provide clear mental health support for all employees through means of occupational therapy, reasonable adjustments and/or internal mental health resources.

## Supporting information

S1 Table Table of supplementary quotes. (DOCX)

S1 File Masking survey: Experiences of workplace masking. (DOCX)
