# Supplementary material for: The workplace masking experiences of autistic, non-autistic neurodivergent and neurotypical adults in the UK
Source: PLoS One. 2023 Sep 6;18(9):e0290001. doi: 10.1371/journal.pone.0290001 (PMC10482295; doi:10.1371/journal.pone.0290001)
Supplement: S1 Table — (DOCX) [file pone.0290001.s001.docx]

**The workplace masking experiences of autistic, neurodivergent and neurotypical adults in the UK.**

Journal of Autism and Developmental Disorders

Amber Pryke-Hobbes^1^, Jade Davies^1^, Brett Heasman^2^, Adam Livesey^1^, Amy Walker^3^, Elizabeth Pellicano^4^, Anna Remington^1^

^1^ UCL Centre for Research in Autism and Education (CRAE), University College London, London, United Kingdom

^2^ School of Education, Language and Psychology, York St John University, York, United Kingdom

^3^ Neurodiversity Works, London, United Kingdom

^4^ Macquarie School of Education, Macquarie University, Sydney, Australia

Correspondence relating to this article should be addressed to Amber Pryke-Hobbes at [a.pryke-hobbes@ucl.ac.uk](mailto:a.pryke-hobbes@ucl.ac.uk)

| **Supplementary Materials 2: Table of Supplementary Quotes** | | | | |
| --- | --- | --- | --- | --- |
|  |  |  | | |
| **Themes** | **Sub-themes** | **Illustrative Quotes** | | |
|  |  | **Autistic participants** | **Neurodivergent participants** | **Neurotypical participants** |
| 1. The value of openness | *1.1 “A role model for difference”* | “In future, I would like to think I could avoid masking continuously, to accentuate/amplify my autistic strengths and differences, to be a role model for difference, diversity & inclusion” | N/A | N/A |
|  | *1.2 The freedom that diagnosis affords* | Having a diagnosis enabled me to be free of this, at least with the close team I've disclosed to, and I think it's made me happier at work” | “I honestly don’t really bother to mask - getting a diagnosis gave me permission to e.g. wear comfy shoes, stop making eye contact if it’s uncomfortable etc.” | N/A |
|  | *1.3 A genuine acceptance of difference* | “I find it quite hard fitting into a mainstream workplace where people aren't always willing to make the time to be patient about my autism and the lack of clarity in myself perceiving certain social situations, most often when I take things personally. I can't always feel like myself around people who expect more of me or who don't understand my difficulties as well as other people who love and support me at heart.” | “In other areas of life people are more accepting as they are not financially impacted so it is easier to be open.” | “My wife and family accept me, but with work and friends I am more careful” |
| 2. A desire to socially integrate | *2.1 Fitting in with the norm* | “I tried incredibly hard to create a self that was like other people and got quite good at it.” | “To fit in , to be perceived in ways that are "acceptable" i.e. team player, extroverted, friendly.” | “In the workplace you mask to try to fit in” |
|  | *2.2 Connecting with others* | “Non-autistic people won't want to get close to me, either personally or professionally, if I don't mask as best I can. I think I would be lonelier if I didn't mask.” | “[Workplace masking] is more important to me I don't want to ruin social relationships (though I feel I may have done that already).” | “Being liked, popular [and] engaging [are advantages of masking]” |
|  | *2.3 An attempt to avoid ostracism* | “The advantages are that people cannot detect that I have autism. If they find out I have autism, they will ostracise me” | “I always felt like an outsider and struggled to fit in.” | N/A |
| 3. Avoiding discriminatory and prejudicial treatment | *3.1 A matter of personal safety* | “While masking, I have witnessed how people treat those that are different to them (e.g. having an apparent sound sensitivity, being socially awkward) with malicious gossip or bullying.” | “[consequences of not masking include] abuse, bullying” | “The more noticeable you are the more open to questions or even bullying you are” |
|  | *3.2 Others’ negative perceptions* | “Although I work in an environment which is less judgmental than the general public and more aware that difference is not deficiency, I still feel if I truly never masked I would be perceived as weird” | “There are people who still have perceptions about mental health and therefore, masking it from those people may reduce negative impacts.” | “I worry that I will be judged [if I don’t mask]” |
|  | *3.3 To be treated as an equal* | “The advantages are no one treats me differently. I get invited places and I'm talked to like everyone else.” | “Advantages: Didn’t feel I was being treated differently, wanted to be judged on the same terms as neurotypical people, didn’t receive uninformed comments like others in my workplace, my skills were attributed to hard work and intelligence.” | “[Masking] has meant I'm seen just as me” |
|  | *3.4 An insufficient understanding* | “Employers and colleagues have a poor understanding of my autistic needs, so it is better to hide them as much as possible.” | “Masking just makes things easier when dealing with certain people, trying to explain dyspraxia to them would just be additional aggravation. Especially as it's not as well known as, for example, dyslexia.” | N/A |
| 4. Better employment outcomes | *4.1 Obtaining and maintaining employment* | “If I couldn't mask I wouldn't have a job. I was denied disability support and my family don't support me. My life depends on my ability to mask. Without it I would be unemployed and homeless.” | “the pressure you are under to almost conform and fit in as the risks of being different can effectively change your life as they are linked to your income.” | “advantages [include that] I've stayed in employment for a long time” |
|  | *4.2 Fulfilling professional responsibilities* | “I literally have to mask every single second I am working! I have to speak to every person on the phone as though I am a 'normal' person who speaks and thinks in a 'normal' way (as well as helping them with their query). Being non-verbal is completely out of the question.” | “I find masking in the professional environment more difficult, but to me it feels more essential. As I work in a professional services environment, we need to provide a service.” | “Wanting to appear professional, wanting to appear certain ways to certain individuals, tailoring my communication depending on the audience” |
|  | *4.3 To be perceived as competent and of value* | “Performing at least some of the expected behaviours seems to be essential to be accepted as competent or appropriate, even when they have little to do with the job.” | “Not much different, just more pressure to "keep up appearances" in the workplace as stricter, professional environment where progress is measured by fitting in and by behavior, not just by work ethic or output” | “Wanting to be a respected and trusted member of the team” |
|  | *4.4 “Climbing the ladder”* | “[Masking] is an important element of career progression and if i didn't mask they may not trust me with more responsibility.” | “I mask in the workplace to protect my position. I think disclosing my diagnosis could reduce opportunities [and] slow down career progression.” | “[I mask] to get chartered and promoted quickly and take on more responsibility than others in my grad intake.” |
| 5. Detrimental effects to wellbeing | *5.1 Onerous and exhausting* | “Masking sacrifices my abilities; I hear less, I miss things, I burn more energy, and I cannot use my mind in ways that I know I can do very well, like analysing patterns and rapidly integrating information, because I have to concentrate so hard on looking normal.” | “i found it more exhausting masking in the workplace. at the end of a shift (in any of the jobs i have done) i was so exhausted, confused, frightened, overwhelmed, and anxious etc my head was so full and loud and busy with absolutely everything that had happened … my head felt dizzy, with a high pitched ringing sound, swirling and spinning and a tiredness that was overwhelming” | “Masking is exhausting” |
|  | *5.2 forgoing mental and physical health* | “[Masking] sometimes feels like your treading water and close to drowning or like you can't breathe but the thought of showing people who you really are is scary as well and also makes you panic.” | “disadvantages [include] mental ill health” | “You become overly concerned with how others perceive you - introduces more stress, which in itself becomes something you try to mask. A bit of a vicious circle.” |
|  | *5.3 Worries about getting it wrong* | “there is always the fear that someone will notice and figure out that i am not like them.” | “Disadvantages - I can never truly relax as I am always on edge about how people may perceive my actions. It can cause me to replay situations in my head which I wish had gone better.” | “[it] can be stressful that you fear of being caught out or challenged on your behaviour” |
|  | *5.4 A barrier to support* | “[Masking] hides struggles and creates pressure to always be at the top of your game. Missing out on help and support that might be forthcoming if you're open. People don't believe when you're struggling.” | “By masking the unknown I think it took a lot longer before someone realized that I did have a problem and was able to point me in the right direction to get help.” | “People did not understand how I was feeling [because I was masking]” |
| 6. Presenting an inauthentic self | *6.1 Acting the part* | “I find it easier as there’s a certain predictability in a business environment and with the right knowledge you can basically learn the script for your Role. The social expectations are different (depending on your job I suppose) and language is more formal and less ‘friendly’ which actually suits me quite well. It’s easy to know what to wear, what to say and how to behave in a structured environment” | “I try to act like a successful leader - I don't feel able to be me in a board room” | “Masking allows you to adopt a persona and live through that persona, whether it is close to your true character or not.” |
|  | *6.2 “Feeling like a fraud”* | “If you cannot consistently keep up a mask it can be counterproductive: colleagues have a sense that you are false and/or lacking honesty.” | “on other days [masking] just makes me cringe as it seems so fake.” | “[A] disadvantage [is that you give a] false sense of self character to [the] projectee” |
|  | *6.3 “Not being true to myself”* | “Sometimes after lots of intense masking, the alone-time I need afterwards feels partly about rebuilding myself (or my sense of myself).” | “​​I felt I was looking out of a body that was not mine. In work I had to pretend to be someone who was very far removed from who I believed I was.” | “Disadvantages [include that] I don't really know who I am anymore” |
|  | *6.4 “No one really knows you”* | “it prevents me from experiencing genuine acceptance or authentic relationships within the workplace.” | “as some people in my life only see me masking so I sometimes feel people don't know me as well as they think they do.” | “People don’t get to see the real you” |
| 7. A unique situation | *7.1 A lack of choice* | “In other areas of life, like say at a family party- realistically you can step outside if you need a break from sensory overwhelm. You can leave an optional social event at any time.” | “I can choose my friends. I can't choose some of the people I have to work with” | “In social life, you can choose who to be around, and choose to be around people who accept you; in the workplace you cannot choose your colleagues and as such have to get along with them as best as possible to make life easier.” |
|  | *7.2 Exclusivity of workplace masking* | “It’s not who I am I only do this at work and places a major amount of stress and pressure on me. Outside I am who I am.” | “I don't mask in my social life, if people can't accept you the way you are I don't want to interact with them socially.” | “[I] don't feel the need to mask in my social life” |
| 8. A widespread phenomenon | *8.1 Contextual indifference* | “It isn't [different] really, it's always playing a part, pretending to be neurotypical, remembering to keep eye contact, finding ways around my inability to hear with background noise, keeping a straight face when I'm screaming inside.” | “I'm not sure I am any different whether it's in a professional environment or not, [the] same principles apply.” | “I don't think [social masking and workplace masking] are different” |
|  | *8.2 The habitual and often subconscious nature of masking* | “As I was only diagnosed within the last year, I am writing this in a retrospective way, with the new realization that what I have been doing over decades must be masking.” | “It’s an instinct. I don’t know how to be otherwise” | N/A |
