# Supplementary material for: The workplace masking experiences of autistic, non-autistic neurodivergent and neurotypical adults in the UK
Source: PLoS One. 2023 Sep 6;18(9):e0290001. doi: 10.1371/journal.pone.0290001 (PMC10482295; doi:10.1371/journal.pone.0290001)
Supplement: S1 File — (DOCX) [file pone.0290001.s002.docx]

**The workplace masking experiences of autistic, neurodivergent and neurotypical adults in the UK.**

Journal of Autism and Developmental Disorders

Amber Pryke-Hobbes^1^, Jade Davies^1^, Brett Heasman^2^, Adam Livesey^1^, Amy Walker^3^, Elizabeth Pellicano^4^, Anna Remington^1^

^1^ UCL Centre for Research in Autism and Education (CRAE), University College London, London, United Kingdom

^2^ School of Education, Language and Psychology, York St John University, York, United Kingdom

^3^ Neurodiversity Works, London, United Kingdom

^4^ Macquarie School of Education, Macquarie University, Sydney, Australia

Correspondence relating to this article should be addressed to Amber Pryke-Hobbes at [a.pryke-hobbes@ucl.ac.uk](mailto:a.pryke-hobbes@ucl.ac.uk)

**Supplementary Materials 1: Masking survey Questions**

**Experiences of masking**

Masking is a term that describes the strategies people use to fit in at their workplace. For example, strategies might apply to the way people manage their appearance, interact and control their own instinctive behaviours. In this section, if you feel comfortable to do so, please share your experiences of masking in the workplace.

1. **What are your motivations for masking or not in the workplace?**

[open text]

1. **How is masking in the workplace different from masking in other areas of social life?**

[open text]

1. **What are the advantages and disadvantages of masking in your experience?**

[open text]
